# Supplementary material for: Indiscriminate activities of different henipavirus polymerase complex proteins allow for efficient minigenome replication in hybrid systems
Source: J Virol. 2024 May 23;98(6):e00503-24. doi: 10.1128/jvi.00503-24 (PMC11237569; doi:10.1128/jvi.00503-24)

SUPPLEMENTAL MATERIALS

Fig S1 Sequence alignment of NiV Bangladesh strain and HeV Redlands strain N and P protein. (**A**) Sequence alignment of N protein of NiV and HeV. Four boxes of N_{tail} are indicated by green dashed-line boxes. Red amino acids represented non-similar residues. Blue amino acids represented similar but non-identical residues. (**B**) Sequence alignment of NiV and HeV P protein. PMD and P_{XD} are indicated by orange dashed-line box. Red text indicates non-similar residues. Blue text represents similar but non-identical residues.

Fig S2 Sequence alignment of the N protein Box 3 domain of Henipaviruses. N protein sequences were downloaded from NCBI virus website. Box 3 regions of (**A**) NiV and (**B**) HeV are aligned. Identical amino acids are indicated by dots, and different amino acids are showed in color. The first and last amino acids positions based on the sequence of NiV Bangladesh strain and HeV Redlands strain are displayed at the top of the figure.

Fig S3 Sequence alignment of PMD of Henipaviruses. PMD sequences of (**A**) NiV and (**B**) HeV were obtained from NCBI virus website and aligned. Identical amino acids are indicated by dots. Different amino acids are showed in color. The first and last amino acids positions based on the sequence of NiV Bangladesh strain and HeV Redlands strain are displayed at the top of the figure.

Fig S4 Sequence alignment of the P_{XD} Henipaviruses. Sequences of (**A**) NiV and (**B**) HeV P_{XD} were obtained from NCBI virus website and aligned. Identical amino acids are indicated by dots. Non-identical amino acids are showed in color. The numbers displayed at the top of the figure are indicated the first and last amino acids positions based on the sequence of NiV Bangladesh strain and HeV Redlands strain sequence.

Fig S5 Sequence alignment of L protein CR I of Henipaviruses. L protein sequences of (**A**) NiV and (**B**) HeV CR I were downloaded from NCBI virus website and aligned. Identical amino acids are indicated by dots. Non-identical amino acids are showed in color. The numbers displayed at the top of the figure

are indicated the first and last amino acids positions based on the sequence of NiV Bangladesh strain and HeV Redlands strain sequence.

Fig. S1

A

```
Nipah N protein    1   MSDIFEEAASFRSYQSKLGRDGRASAATATLTTKIRIFVPATNSPELRWELTLFALDVIRSPSAAESMKVGAAFTLISMY  80
Hendra N protein   1   MSDIFEEAASFRSYQSKLGRDGRASAATATLTTKIRIFVPATNSPELRWELTLFALDVIRSPSAAESMKIGAAFTLISMY  80

Nipah N protein    81  SERPGALIRSLLNDPDIEAVIIDVGSMVNGIPVMERRGDKAQEEMEGLMRILKTARDSSKGKTPFVDSRAYGLRITDMST  160
Hendra N protein   81  SERPGALIRSLLNDPDIEAVIIDVGSMLNGIPVMERRGDKAQEEMEGLMRILKTARESSKGKTPFVDSRAYGLRITDMST  160

Nipah N protein    161 LVSAVITIEAQIWILIAKAVTAPDTAEDSETRRWAKYVQQKRVNPFFALTQQWLTEMRNLLSQSLSVRKFMVEILIEVKK  240
Hendra N protein   161 LVSAVITIEAQIWILIAKAVTAPDTAEESETRRWAKYVQQKRVNPFFALTQQWLTEMRNLLSQSLSVRKFMVEILMEVKK  240

Nipah N protein    241 GGSAKGRAVEIISDIGNYVEETGMAGFFATIRFGLETRYPALALNEFQSDLNTIKSLMLLYREIGPRAPYMVLLEESIQT  320
Hendra N protein   241 GGSAKGRAVEIISDIGNYVEETGMAGFFATIRFGLETRYPALALNEFQSDLNTIKGLMLLYREIGPRAPYMVLLEESIQT  320

Nipah N protein    321 KFAPGGYPLLWSFAMGVATTIDRSMGALNINRGYLEPMYFRLGQKSARHHAGGIDQNMANRLGLSSNQVAELAAAVQETS  400
Hendra N protein   321 KFAPGGYPLLWSFAMGVATTIDRSMGALNINRGYLEPMYFRLGQKSARHHAGGIDQNMANKLGLNSDQVAELAAAVQETS  400

Nipah N protein    401 AGRQESNVQAREAKFAAGGVLIGGSDQDVDEEEEPIEQSGRQSVTFKREMSISSLADSVPSSSVSTSGGTRLTNSLLNLR  480
Hendra N protein   401 VGRQDNNMQAREAKFAAGGVLVGGGEQDIDEEEEPIEHSGRQSVTFKREMSMSSLADSVPSSSVSTSGGTRLTNSLLNLR  480

Nipah N protein    481 SRLAAKAAKEAASSNATDDPAISNKDQRESEKKNNQDLKPTQNDLDFVRADV*  533
Hendra N protein   481 SRLAAKAIKESTAQSSSGRNPPNNRPQADSGEKDDQESKPAQNDLDFVRADV*  533
```

Box 1 Box 2 Box 3 Box 4

B

```
Nipah P protein    1   MDKLELVNDGLNIIDFIQKNQKEIQKTYGRSSIQQPSIKDRTKAWEDFLQCTSGESEQVEGGMSKDDGGVERRSLEDLSS  80
Hendra P protein   1   MDKLDLVNDGLDIIDFIQKNQKEIQKTYGRSSIQQPSTKDRTRAWEDFLQSTSGEHEQAEGGMPKNDGGTEGRNVEDLSS  80

Nipah P protein    81  TSPTDGTIGKRVSNTRDWAEGSDDIQLDPVVTDVVYHDHGGECTGYGFTSSPERGWSDHSSGANNGDVCLVSDAKVLSYA  160
Hendra P protein   81  VTSSDGTIGQRVSNTRAWAEDPDDIQLDPMVTDVVYHDHGGECTGHGPSSSPERGWSYHMSGTHDGNVRAVPDTKVLPNA  160

Nipah P protein    161 PEIAVSKEDRETDLVHLEDKLSATGLNPTAIPFTPKNLSVPAKDSPVIAEHYYGLGVREQNVDPQTNRNVNLDSIKLYTS  240
Hendra P protein   161 PKTTVPEEVGEIDLIGLEDKFASAGLNPAAVPFVPKNQSTPTEEPPVIPEYYYGSGRRGDLSKSPPRGNVNLDSIKIYTS  240

Nipah P protein    241 DDEEADQLEFEDEFAGSSSEVIVGISPEEE----------EPSSAGRKPIESVGHIIEGQST-----RDSLQIKGNKPAD  305
Hendra P protein   241 DDEDENQLEYEDEFAKSSSEVVIDTTPEDNDSINQEEVVGDPSDQGLEHPFPLGKFPEKEETPDVRRKDSLM--------  312

Nipah P protein    306 APGAGPKDSAVKEKSPQKRLPMLAEEFECSGSEDPIIQELLKENSFINSQQGKDAQPLYYRGIEGSRSPDKTEITSDAVQ  385
Hendra P protein   312 ------QDSCKREGVP-KRLPMLSEEFECSGSDDPIIQELEREGSHPGGSL-RLREPPQSSG-NSRNQPDRQLKTGDAAS  383

Nipah P protein    386 TANKQRPGTPMPKSRGIPIKKGTDEKYPSAGTENVPGSKSGATRHVRGSPPYQEGKSVNAENVQLNVPTVVKETDKSEAN  465
Hendra P protein   384 PGGVQRPGTPMPKSRIMPIKKGTDAKSQYVGTEDVPGSKSGATRYVRGLPPNQESKSVTAENVQLSAPSAVTRNEGHDQE  463

Nipah P protein    466 PADDNDSLDDKYIMPSDDFSNTFFPHDTDRLNYHADHLGDYDLETLCEESVLMGVINSIKLINLDMRLNHIEEQVKEIPK  545
Hendra P protein   464 VTSNEDSLDDKYIMPSDDFANTFLPHDTDRLNYHADHLNDYDLETLCEESVLMGIVNAIKLINIDMRLNHIEEQMKEIPK  543

Nipah P protein    546 IINKLESIDRVLAKTNTALSTIEGHLVSMMIMIPGKGKGERKGKSNPELKPVIGRDVLEQQSLFSFDNVKNFRDGSLTNE  625
Hendra P protein   544 IINKIDSIDRVLAKTNTALSTIEGHLVSMMIMIPGKGKGERKGKTNPELKPVIGRNILEQQELFSFDNLKNFRDGSLTDE  623

Nipah P protein    626 PYGAAVQLRGDLILPELNFEETNASQFVPMADDSSRDVVKTLIRTHIKDRELRSELIGYLNRAENDEEIQEIANTVNDII  705
Hendra P protein   624 PYGGVARIRDDLILPELNFSETNASQFVPLADDASKDVVRTMIRTHIKDRELRSELMDYLNRAETDEEVQEVANTVNDII  703

Nipah P protein    706 DGNI*  710
Hendra P protein   704 DGNI*  708
```

PMD P_{XD}

Fig. S2

A

473 – 493

Consensus	TNSLLNLRSRLAAKAAKEAAS
NiV Bangladesh N prot...	....
NP_112021.1	....
WKR82401.1	....
UEK25998.1	....
QHR78949.1	....
QHR78958.1	....
QHR78967.1	....
QHR78976.1	....
QHR78985.1	....
QHR78994.1	....
QHR79003.1	....
QHR79012.1	....
QHR79021.1	....
QHR79030.1	....
QHR79039.1	....
QHR79048.1	....
QHR79057.1	....
QHR79075.1	....
QHR79084.1	....
QHR79093.1	....
QHR79102.1	....
QHR79111.1	....
QHR79120.1	....
QHR79129.1	....
QHR79138.1	....
QHR79147.1	....
QHR79156.1	....
QHR79165.1	....
QHR79174.1	....
QHR79183.1	....
QHR79192.1	....
QHR79201.1	....
QHR79210.1	....
QHR79219.1	....
QHR79228.1	....
QHR79237.1	..................T..
QHR79246.1	....
QPD07335.1	....
QPD07345.1	....
QPD07346.1	....
QPD07347.1	....
QPD07348.1	....
QPD07350.1	....
QPD07356.1	....
QPD07361.1	....
QPD07362.1	....
QPD07363.1	....
QKV44007.1	....
QKV44016.1	....
QKV44052.1	....
QKV44061.1	....
QKV44079.1	....
QCY54414.1	....
QCY54423.1	....
QDJ04459.1	....
QCY59029.1	....
QCY59035.1	....
QCY59041.1	....
QCY59047.1	....
QCY59052.1	....
QCY59058.1	....
QCY59064.1	....
QCY59070.1	....
QCY59076.1	....
QCY59082.1	....
QCY59088.1	....
QBQ56698.1	....
QBQ56707.1	....
QBQ56716.1	....
AWT50987.1	....
APT69627.1	....
APT69694.1	....
ALO75942.1	....
ALO75943.1	....
ALO75944.1	....
ALO75945.1	....
ALO75946.1	....
ALO75947.1	....
ALO75948.1	....
ALO75949.1	....
ALO75950.1	....
ALO75951.1	........L............
AIS25032.1	....
AEZ01370.1	....
AEZ01371.1	....
AEZ01372.1	....
AEZ01373.1	....
AEZ01374.1	....
AEZ01384.1	....
AEZ01393.1	....
CBM41030.1	....
ACT32611.1	....
AAX51852.1	....
AAY43911.1	....
CAF25493.1	....
CAD92347.1	....
CAD92353.1	....
CAD92359.1	....
AAM13401.1	....
AAK50540.1	....
AAK50548.1	....
AAF73377.1	....

B

473 – 493

Consensus	TNSLLNLRSRLAAKAIKESTA
HeV Redlands N protein	....
NP_047106.1	....
UCY33663.1	...............M.DNA.
UCY33672.1	...............M.DNA.
UCY33681.1	...............M.DNA.
UCY33690.1	...............M.DNA.
QYC64598.1	...............M.DNA.
QMU23819.1	....
QMU23820.1	....
QMU23822.1	....
QDK64763.1	....
APT69524.1	....
AEQ38019.1	....
AEQ38028.1	....
AEQ38037.1	....
AEQ38046.1	....
AEQ38055.1	....
AEQ38064.1	....
AEQ38073.1	....
AEQ38092.1	....
AEQ38101.1	....
AEQ38109.1	....
AEQ38117.1	....
AEQ38126.1	....
AEQ38134.1	....
AEB21191.1	....
AEB21200.1	....
AEB21209.1	....
AEB21218.1	....
AEB21227.1	....
O89339.1	....
AAC83187.1	....

Fig. S3

A

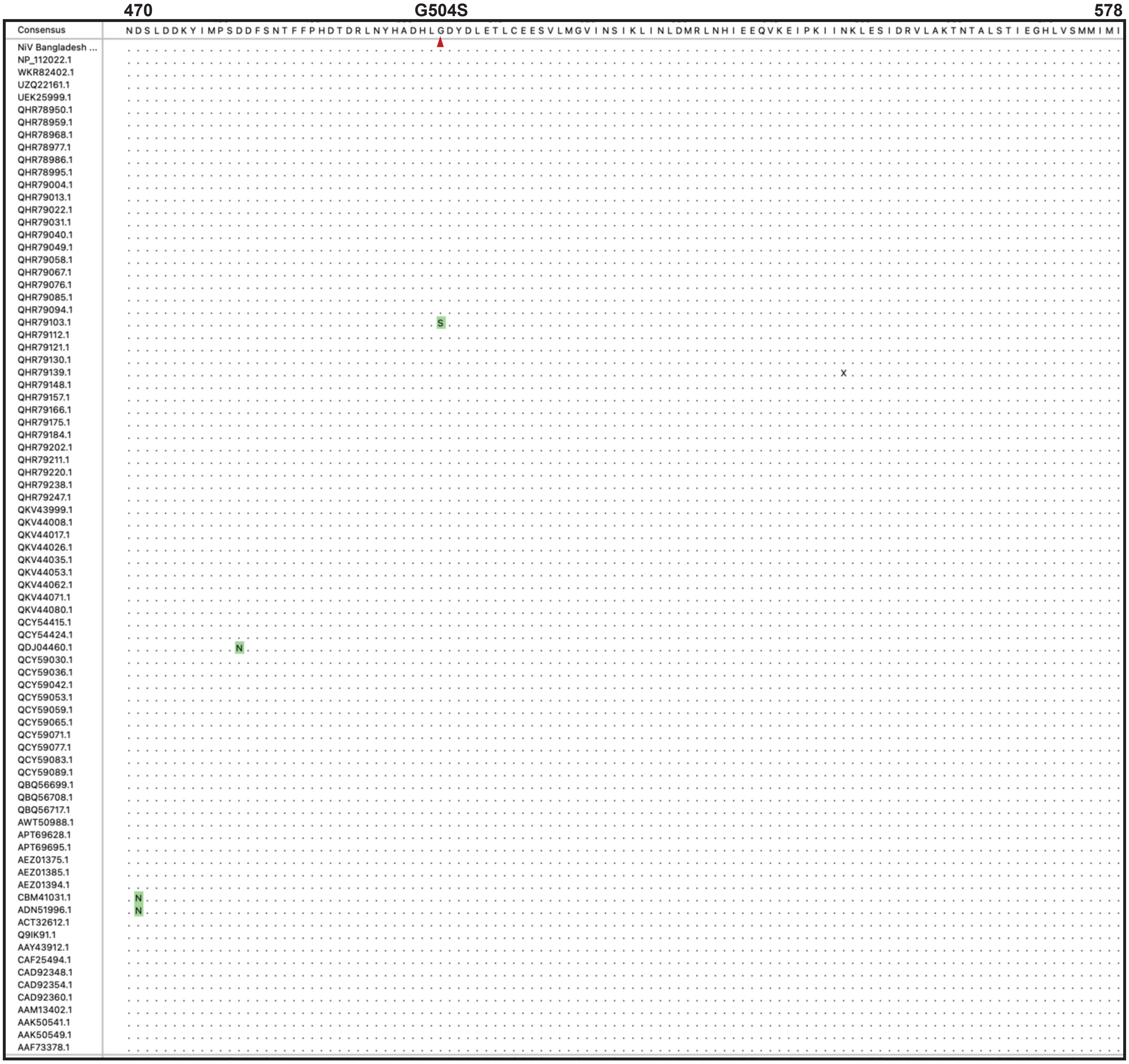

B

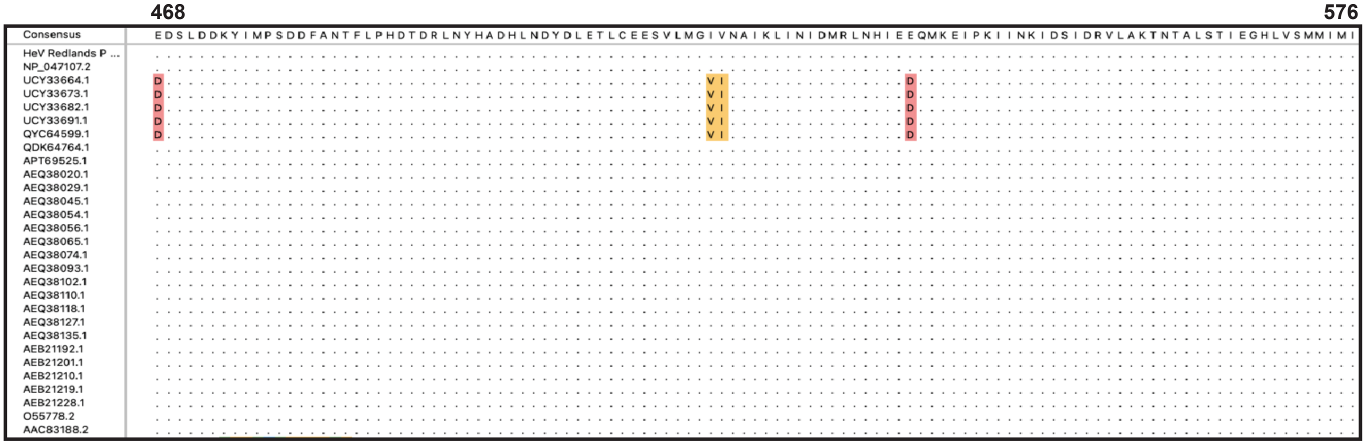

Fig. S4

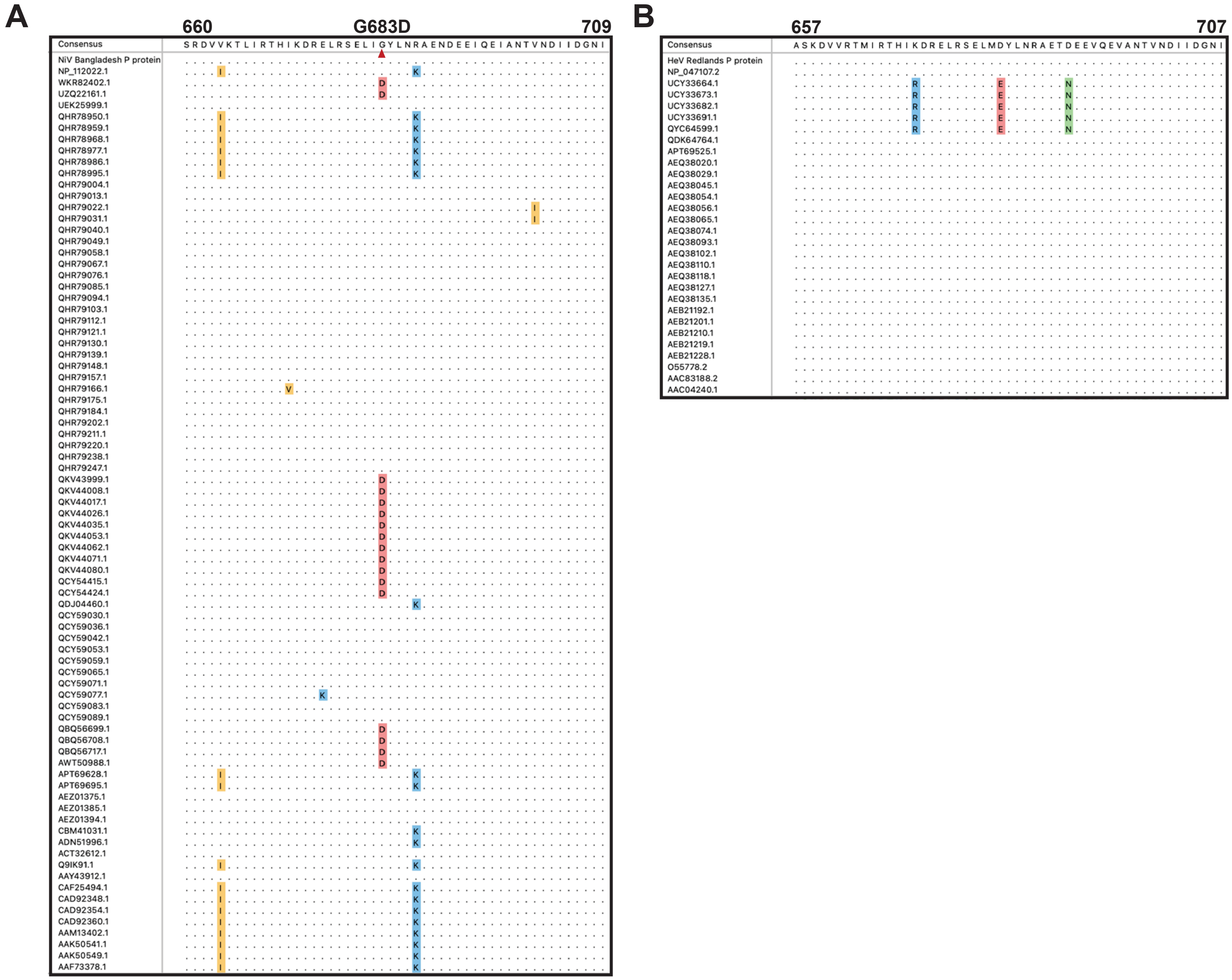
A
660
G683D
709
Consensus
S R D V V K T L I R T H I K D R E L R S E L I G Y L N R A E N D E E I Q E I A N T V N D I I D G N I
NiV Bangladesh P protein
B
657
707
Consensus
A S K D V V R T M I R T H I K D R E L R S E L M D Y L N R A E T D E E V Q E V A N T V N D I I D G N I
HeV Redlands P protein

Fig. S5

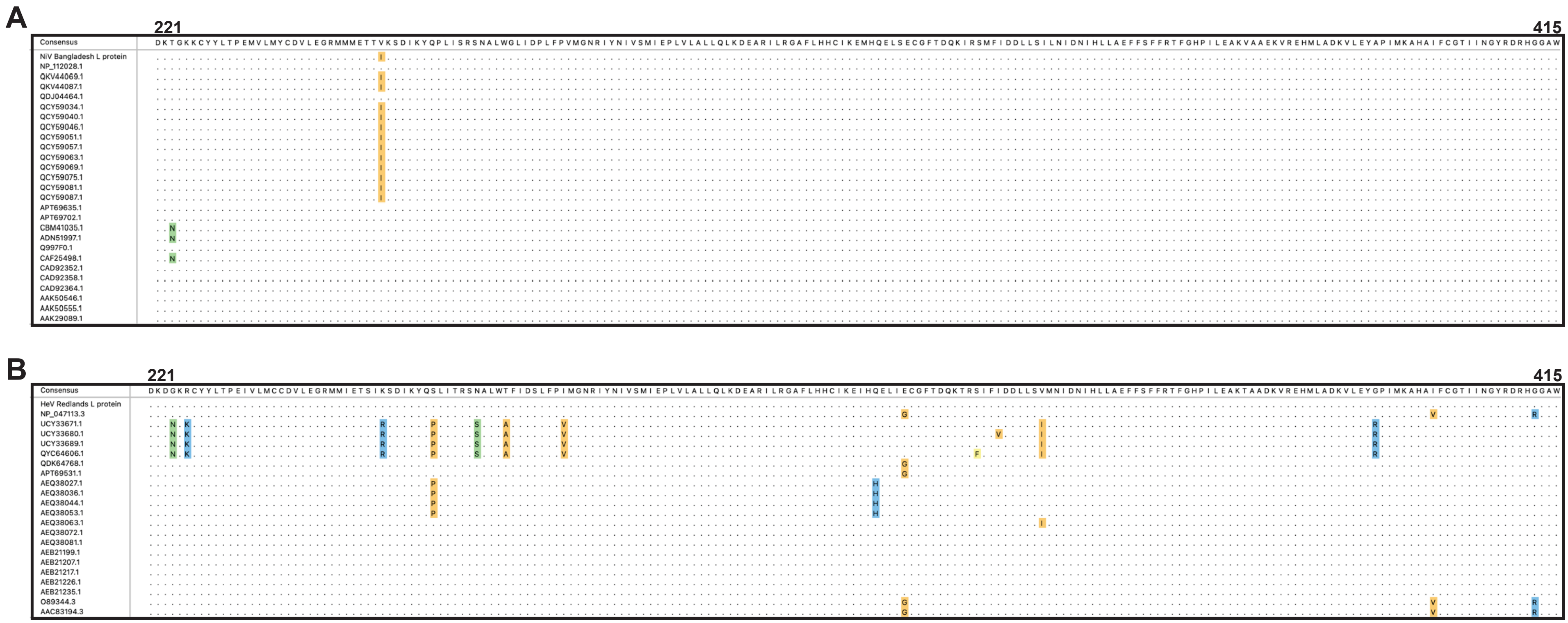

Supplement: Fig. S1 to S5 — Sequence analysis of henipavirus N, P, and L proteins. [file jvi.00503-24-s0001.pdf]
